# Antiferromagnetic Arsenides U_8_Co_42_As_25_ and UCo_3_As_2_

**DOI:** 10.1021/acs.inorgchem.6c01592

**Published:** 2026-07-11

**Authors:** Nazar Zaremba, Mitja Krnel, Yurii Prots, Orest Pavlosiuk, Lev Akselrud, Andreas Leithe-Jasper, Markus König, Yuri Grin, Eteri Svanidze

**Affiliations:** † Max Planck Institute for Chemical Physics of Solids, 01187 Dresden, Germany; ‡ 28270 Institute of Low Temperature and Structure Research, Polish Academy of Sciences, 50-422 Wrocław, Poland; § Department of Inorganic Chemistry, Ivan Franko National University of Lviv, 79000 Lviv, Ukraine

## Abstract

Arsenides continue to captivate the fields of inorganic chemistry and condensed matter physics. In this work we present the discovery of two new members of this family—U_8_Co_42_As_25_ and UCo_3_As_2_—which were synthesized in single crystalline form. U_8_Co_42_As_25_ is the first representative of the Y_8_Co_41_As_25_structure type (space group *P*6_3_/*m*, Pearson symbol *hP*75, *a* = 17.7460(5) Å, *c* = 3.8120(1) Å), while UCo_3_As_2_ adopts HoCo_3_P_2_ type of structure (space group *Pmmn*, Pearson symbol *oP*36, *a* = 3.8611(2) Å, *b* = 10.8414(6) Å, *c* = 12.7297(6) Å). Both materials order antiferromagnetically—U_8_Co_42_As_25_ at *T*_*N*_ = 26 K and UCo_3_As_2_ at *T*_*N*_ = 67 K.

## Graphical abstract

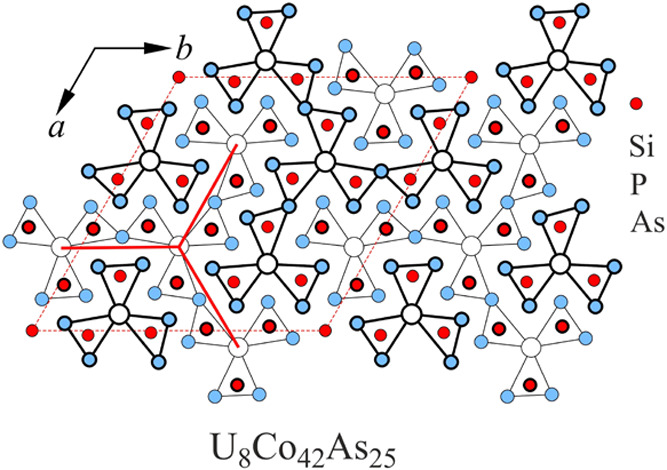

## 1 Introduction

Uranium-based materials remain significantly less explored, compared to their rare-earth analogues—mostly as a result of their fragile ground states coupled with nontrivial synthetic requirements. Yet, a number of exciting chemical and physical phenomena have been recently attributed to the peculiarity of *f*-electrons, making such explorations both relevant and timely. In the past few years, a plethora of emergent behavior in both magnetic and superconducting materials based on uranium has been reported—from spin-triplet superconductivity to complex magnetic orders, topological surface states to multipolar orders.^1–8^

Among binary U–Co and U–As compounds, both superconductivity and magnetic ordering are found. The most uranium-rich uranium–cobalt binary U_6_Co material displays phonon-mediated superconductivity below *T*_*c*_ = 2.4 K.^9^ The UCo, UCo_2_ ^10,11^ and UCo_3_ ^12^ compounds are paramagnets, while the most cobalt-rich compound UCo_5.3_ ^13,14^ orders ferromagnetically above room temperature (*T_c_* = 360 K). In the U–As binary, UAs^15^ and UAs_2_ ^16^ show antiferromagnetic transitions at 126 and 283 K, respectively, while U_3_As_4_ ^17^ is a ferromagnet below *T*_*c*_ = 198 K. Within the Co–As binary, both CoAs_2_ ^18^ and CoAs_3_ ^19^ are diamagnets, Co_2_As^20^ is ferrimagnetic below 60 K, while CoAs is a paramagnetic material. A natural extension into the U–Co–As phase space was successful in discovering two compounds – UCoAs_2_ ^21^ and U_2_Co_12_As_7_,^22^ the first one exhibits ferromagnetic ordering at 144 K. Since more compositions are known for the *R*–Co–As system—*R*Co_2_As_2_ (*R* = La–Nd, Eu),^23–29^ *R*_2_Co_12_As_7_ (*R* = Y, Pr, Nd, Sm–Er, and Yb),^22,30,31^ and *R*Co_5_As_3_ (*R* = Gd–Er)^32,33^ – it is reasonable to assume that more compounds can be found in the U–Co–As system.

The search for new materials within the U–Co–As phase diagram prompted our investigation. We concentrated on the Co-rich part of this system, and in this work we report the discovery of two new arsenides – U_8_Co_42_As_25_ and UCo_3_As_2_. Both materials belong to a large group of ternary intermetallic compounds with a metal–metalloid ratio of 2:1. They order antiferromagnetically with signs of magnetic frustration as a result of the triangular motifs in their structure.

## 2 Experimental Details

Due to the toxicity of constituent elements, all sample preparation and handling were performed in the specialized laboratory, equipped with an argon-filled glovebox system (MBraun, p­(H_2_O/O_2_) < 0.1 ppm).^34^ Single crystals of U_8_Co_42_As_25_ and UCo_3_As_2_ were grown from the bismuth flux. Pure elements of U (wire, Goodfellow, 99.98%), Co (powder, ChemPur, 99.9%), As (pieces, Puratronic, 99.999%), and Bi (granules, ChemPur, 99.999%) in the ratios 1:5:3:20 and 1:3:2:20 were placed in an alumina Canfield-Svanidze crucible set^35^ and subsequently sealed in a Ta tube. Each sample was heated to 1150 °C for 48 h, held at this temperature for 12 h, and cooled to 700 °C for 168 h with further cooling to room temperature over 48 h in a vertical furnace. The tantalum tube with the mixture was then sealed in the silica tube, heated to 500 °C, and placed into the centrifuge to remove the bismuth flux. The resultant product consisted of shiny gray needle-shaped crystals, which grow along the shortest crystallographic directions [001] (U_8_Co_42_As_25_) and [100] (UCo_3_As_2_), as shown in Figure 1. Unfortunately, their small size was not sufficient for anisotropic measurements of physical properties. Both compounds appear to be stable in air over long periods of time.

**Figure 1:**
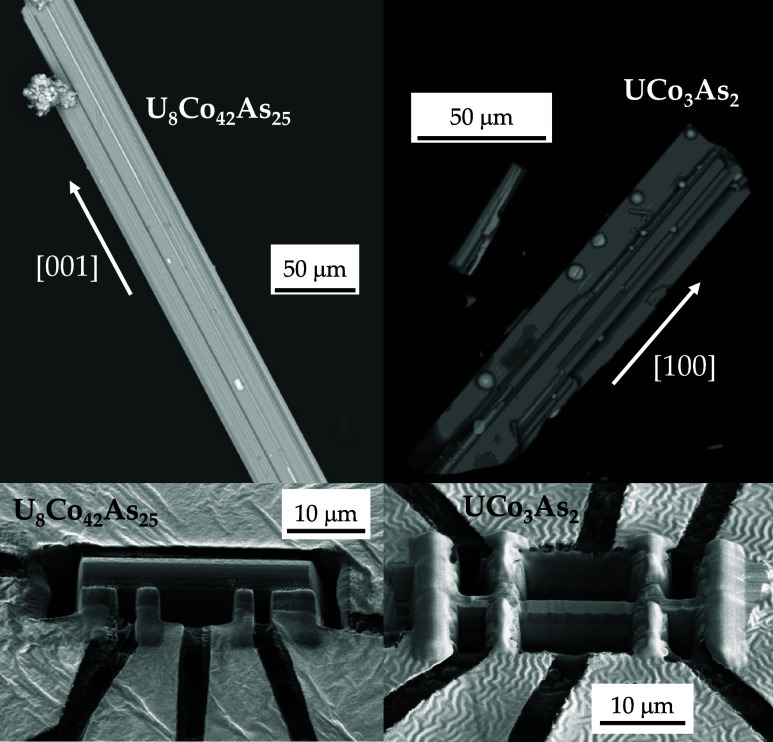
SEM images of U_8_Co_42_As_25_ (left) and UCo_3_As_2_ (right) single crystals, together with the respective microscale devices (bottom). The electrical resistivity is measured along the [001] (U_8_Co_42_As_25_) and [100] (UCo_3_As_2_) directions. Small amount of Bi as droplets or channels is present on the surface of the crystals (light gray color).

Powder X-ray diffraction measurements (see Figures S2 and S3) were performed on a Huber G670 image plate Guinier camera with a Ge-monochromator (Cu Kα_1_ radiation, λ = 1.54056 Å). The phase identification was done using the Match 3! software.^36^ For single-crystal experiments, relatively thin (∼20–30 μm), but long (∼150–250 μm) specimens were used. The diffraction data were collected using a Rigaku AFC7 diffractometer, equipped with a Saturn 724+ CCD detector. The data reduction was performed by using the CrysAlis^*PRO*^SM software.^37^ Empirical absorption correction was done by a multiscan routine.^38^ The WinGX suite of programs^39^ was used for structure solution, refinement, and data analysis.

The crystals of U_8_Co_42_As_25_ and UCo_3_As_2_ were also analyzed by energy-dispersive X-ray spectroscopy with a Jeol JSM 6610 scanning electron microscope, equipped with an UltraDry EDS detector (Thermo Fisher NSS7). The semiquantitative analysis was carried out with a 30 keV acceleration voltage. The experimentally determined element ratio of U:Co:As was 8.0:48.0:29.0 for U_8_Co_42_As_25_ and 1.0:3.4:2.1 for UCo_3_As_2_, which is in good agreement with the compositions obtained from the structure refinement.

Temperature- and field-dependent magnetic measurements were conducted using a Quantum Design (QD) Magnetic Properties Measurement System (MPMS). Several single crystals of either of the compounds were mounted on quartz capillaries. The magnetic moment was measured at temperatures ranging from 2 to 300 K and in magnetic fields up to μ_0_ *H* = 7 T for both zero-field-cooled and field-cooled cases. The specific heat data were collected on a QD Physical Property Measurement System (PPMS) from *T* = 0.4 K to *T* = 300 K in *H* = 0 magnetic field. For measurements of electrical resistivity, microscale devices were fabricated out of U_8_Co_42_As_25_ and UCo_3_As_2_ single crystals by using a plasma focused-ion-beam (FIB).^40^ The details of the procedure of micro device preparation are described elsewhere.^40–43^ AC electrical resistivity measurements were performed on a QD PPMS, using a standard four-probe technique at temperatures between *T* = 2 and 300 K in *H* = 0 magnetic field. A current pulse of 0.01 mA with frequency 93 Hz for 1 s was applied along the [001] (U_8_Co_42_As_25_) and [100] (UCo_3_As_2_) directions.

## 3 Results and Discussion

Analysis of the collected diffraction data for U_8_Co_42_As_25_ did not provide a clear indication of a possible space group. A comparison of intensities of the equivalent reflections for the hexagonal Laue classes 6/*m* (*R*_int_ = 0.040) and 6/*mmm* (*R*_int_ = 0.046) favors the latter, as more symmetrical. However, no solution was found for any space group of this Laue class. The nature of the 6-fold axis parallel to [001], e.g., 6 or 6_3_, was also not evident, due the limited number of 00*l* reflections caused by the short lattice parameter *c* = 3.81 Å. Through trial and error, we first assumed that the phase under investigation could be isostructural to Y_8_Co_42–*x*_As_25_,^44^ given the lattice parameters (*a* = 17.74 Å and *c* = 3.81 Å) and the relative ratios of the elements. This assumption was confirmed by comparing the recorded powder diffraction pattern with the calculated one, obtained using this structure model. Thus, the atomic coordinates of Y_8_Co_41*x*_As_25_ were used as initial values for the refinement of the crystal structure of U_8_Co_42_As_25_. Despite the reliable structural model, the refinement led to unsatisfactory residuals: *R*1 = 0.1699 and *w*R** 2 = 0.3771. Analysis of the refinement protocol revealed features that are typical for twinned crystals:

(a) almost identical *R*_int_ for Laue classes 6/*m* (in which the crystal structure is described) as well as higher symmetrical 6/*mmm*, as mentioned above;
(b) enhanced intensities on the difference Fourier maps close to the existing atomic positions;
(c) systematically, *F*_obs_ are larger than *F*_calc_ for most disagreeable reflections;
(d) poor matching of weak reflections.

By applying the twinning law (110 010 001), which simulates a mirror plane perpendicular to [010], the residuals were reduced to *R*1 = 0.0373 and *w*R** 2 = 0.0708. Particular attention should be paid to the part of the structure along [001]. As is usual for such a family of compounds, the position of As5, located at site 2*a*, was refined as half-occupied. This is caused by the short lattice parameter *c* = 3.81 Å, which makes it impossible for two atoms to be located at a small distance of about 1.91 Å. Thus, only one of the positions 0 0 1/4 or 0 0 3/4 remains occupied. As a result, the next atoms (in our case, Co2) which are in contact with As5 shift toward [001] or away from it, depending on whether an As atom is present or not. Such a scenario is usually described as a single position for neighboring atoms with increased displacement parameters or, alternatively, as a split position. We prefer the latter, because this model is better for describing the real local situation in the structure and interpretation of interatomic distances. This issue was discussed in detail in our recent publication.^45^ The implementation of the split positions Co2a and Co2b, the occupancies of which were constrained to that of the As5 site, resulted in almost identical occupancy of 0.516(5) and 0.484, respectively. Thus, in each channel along [001], As5 atoms occupy every second position of the crystallographic 2*a* site. Consequently, the Co atoms shift toward or away from the [001] axis, which is reflected in the distances of 1.75 and 2.27 Å between the vacant and occupied by As position (Figure 2a). The latter value is comparable to the shortest As–Co contacts observed at other sites in the structure (Table S4). At the same time, the residuals were reduced to *R*1 = 0.0337 and *w*R** 2 = 0.0628. The observed disorder does not affect the U coordination in the U_8_Co_42_As_25_ structure.

**Figure 2:**
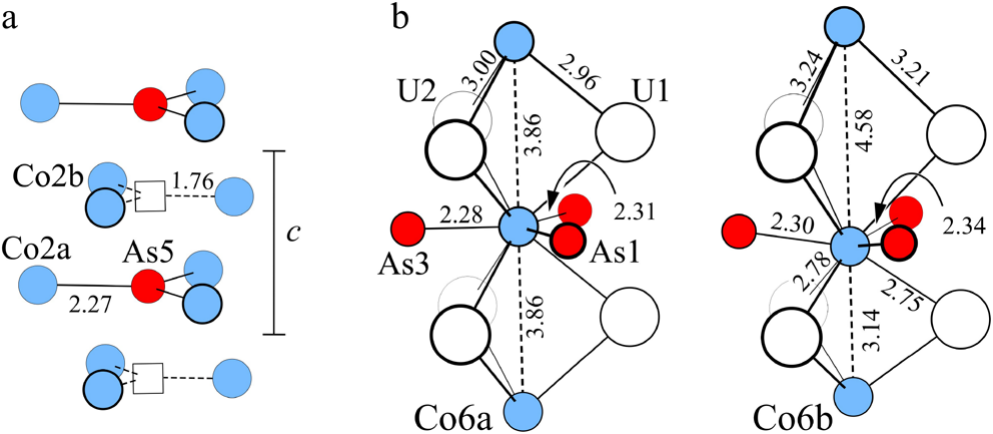
(a) Distribution of As atoms at the 2*a* site in the structure of U_8_Co_42_As_25_, showing the interatomic distances between the Co atoms and the occupied and unoccupied positions. Unoccupied positions are marked with open squares. (b) The local environment of the Co6a and Co6b atoms in the structure of UCo_3_As_2_.

It should be noted that the refinement of the U_8_Co_42_As_25_ structure was carried out using the averaged intensities of the equivalent reflections (Laue class 6/*m*). Normally, when refining twins, the intensities of the equivalent reflexes are not averaged. In this case, however, this is possible because in this merohedral twin, every set of symmetrically equivalent reflections for one domain coexists with only one set of symmetrically equivalent reflections of the second domain, e.g., {120} reflections of one twin coexist with {210} reflections of the second twin, etc. To verify the correctness of this assumption, in separate runs, the refinement was carried out without averaging the reflections, resulting in an identical model with comparable residuals of *R*1 = 0.0404 and *w*R** 2 = 0.0814 for 18463 observed and 20359 measured reflections, respectively. The final refinement was carried out using the averaged intensities (Table 1).

**Table 1:** Crystallographic Data of U_8_Co_42_As_25_ and UCo_3_As_2_ ^a^ ^,^ ^b^

Composition	U_8_Co_42_As_25_ ^c^	UCo_3_As_2_
Structure type	Y_8_Co_41_As_25_	HoCo_3_P_2_
Space group	*P*6_3_/*m*	*Pmmn*
*Z*	1	6
Pearson symbol	*hP*75	*oP*36
Lattice parameters		
*a*/Å	17.7460(5)	3.8611(2)
*b*/Å	*a*	10.8414(5)
*c*/Å	3.8120(1)	12.7297(6)
*V*/Å^3^	1039.64(6)	532.86(4)
Calc. density/g cm^–3^	9.986	10.558
Radiation	Mo K α, λ = 0.71073 Å	
Absorption coeff./mm^–1^	66.999	77.435
Absorption correction	multiscan	
*T*(max)/*T*(min)	1.81	3.3
2θ range (°)	4.6–82.08	6.4–81.98
Range in *h*, *k*, *l*	–32 ≤ *h* ≤ 27	–4 ≤ *h* ≤ 7
	–31 ≤ *k* ≤ 32	–20 ≤ *k* ≤ 16
	–2 ≤ *l* ≤ 6	–18 ≤ *l* ≤ 23
*R*_int_	0.0399	0.0647
*N*(*hkl*) measured	20365	10513
*N*(*hkl*) unique	2517	2002
*N*(*hkl*) observed	2460	1842
Observation criterion	*F(hkl)* ≥ 4σ [*F(hkl)*]	
Refined parameters	79	63
*R*1	0.0337	0.0411
*w*R** 2	0.0629	0.0743
Residual peaks/e Å^–3^	3.19/–3.69	4.73/–4.47

a Although the composition of the latter structure differs from that of the structure described in terms of partial occupation, we regard both structures as isotypical, as they are described by the same atomic motif by the same set of atomic positions with the same space group.

b The .cif files have been deposited at the Cambridge Crystallographic Data Centre (CSD numbers 2540944 and 2540955) and contains the supplementary crystallographic data for this paper. These data can be obtained free of charge via uri https://www.ccdc.cam.ac.uk/data_request/cif website, by emailing mailto: data_request@ccdc.cam.ac.uk, or by contacting the Cambridge Crystallographic Data Centre, 12 Union Road, Cambridge CB2 1EZ, UK; fax: + 44 1223 336033.

c Refined using twinned specimen; ratio of twin components: 0.540(1):0.460.

The structural model of UCo_3_As_2_ was obtained using direct methods (SIR2014)^46^ in the space group *Pmmn*. Refinement of the initial model with 2U, 6Co, and 4As positions yielded acceptable residuals, *R*1 = 0.041 and *w*R** 2 = 0.0765. While most positions exhibited nearly identical and realistic displacement parameters around *U*_eq_ = 0.007 Å^2^, the Co6 position showed an increased value of *U*_eq_ = 0.0237 Å^2^. The refinement of the occupancy parameter did not result in any deviation from the unit value. The refinement of the current position with isotropic thermal parameter resulted in a pronounced peak on the difference Fourier maps of ∼22 e Å^–3^ near this position outside the ( $\frac{1}{4}$ 0 0) plane. The final runs were performed with these two positions, Co6a and Co6b, and yielded an occupancy ratio of 0.57(4):0.43. In case of UCo_3_As_2_, the observed disorder also influences the environment of the U atom (see Figure 2b). In separate runs, a possible structural model was examined for the noncentrosymmetric space group *P*2_1_ *mn*, but this did not lead to any improvement in the description of the structure or an essential reduction of residuals.

In both cases, the structural models were standardized using the STRUCTURE TIDY program.^47^ The complete crystallographic information and atomic coordinates with equivalent displacement parameters are summarized in Tables 1, 2 and 3. The anisotropic displacement parameters and interatomic distances are listed in Tables S1–S4.

**Table 2:** Atomic Coordinates and Equivalent Displacement Parameters (in Å^2^) in the U_8_Co_42_As_25_ ^a^

atom	Wyckoff site	*x*/*a*	*y*/*b*	*z*/*c*	*U*_eq/iso_
U1	6*h*	0.26336(2)	0.32918(2)	1/4	0.00804(5)
U2	2*d*	1/3	2/3	3/4	0.00797(8)
Co1	6*h*	0.04402(7)	0.55518(7)	1/4	0.00779(15)
Co2a^a^	6*h*	0.08401(19)	0.1087(2)	1/4	0.0083(5)
Co2b^a^	6*h*	0.10733(15)	0.14101(17)	1/4	0.0136(5)
Co3	6*h*	0.09861(7)	0.35178(8)	1/4	0.00914(17)
Co4	6*h*	0.24588(8)	0.50748(7)	1/4	0.00829(18)
Co5	6*h*	0.25195(7)	0.13797(8)	1/4	0.00903(16)
Co6	6*h*	0.49167(7)	0.14758(8)	1/4	0.00743(17)
Co7	6*h*	0.49220(8)	0.36946(7)	1/4	0.01130(19)
As1	6*h*	0.00782(5)	0.40923(5)	1/4	0.00732(12)
As2	6*h*	0.01770(5)	0.19891(5)	1/4	0.00805(13)
As3	6*h*	0.18888(5)	0.60150(5)	1/4	0.00736(13)
As4	6*h*	0.40793(5)	0.21586(5)	1/4	0.00763(12)
As5^a^	2*a*	0	0	1/4	0.098(6)

a Occupancies of Co2a, Co2b and As5 were constrained: 0.516(5) and 0.484 for Co2a­(As5) and Co2b, respectively. Co2a and Co2b were refined with isotropic displacement parameters.

**Table 3:** Atomic Coordinates and Equivalent Displacement Parameters (in Å^2^) in the UCo_3_As_2_ ^a^

atom	Wyckoff site	*x*/*a*	*y*/*b*	*z*/*c*	*U*_eq/iso_
U1	2*b*	1/4	3/4	0.52335(3)	0.00707(8)
U2	4*e*	1/4	0.56763(2)	0.79233(2)	0.00674(6)
Co1	2*a*	1/4	1/4	0.84410(12)	0.0114(3)
Co2	2*b*	1/4	3/4	0.98139(11)	0.0068(2)
Co3	4*e*	1/4	0.05249(9)	0.57068(8)	0.00730(16)
Co4	4*e*	1/4	0.08250(9)	0.00387(8)	0.00696(16)
Co5	4*e*	1/4	0.62838(9)	0.29198(8)	0.00768(16)
Co6a^a^	2*a*	1/4	1/4	0.3001(2)	0.0061(4)
Co6b^a^	4*f*	0.157(2)	1/4	0.2996(5)	0.0061(4)
As1	4*e*	1/4	0.06287(6)	0.38614(6)	0.00592(12)
As2	4*e*	1/4	0.59784(7)	0.10831(6)	0.00638(12)
As3	2*a*	1/4	1/4	0.12098(8)	0.00583(16)
As4	2*a*	1/4	1/4	0.65587(8)	0.00671(17)

a Occupancy for Co6a and Co6b atoms are 0.597(12) and 0.202(6) respectively. Isotropic displacement parameter of Co6a and Co6b were costrained U_iso_(Co6a) = U_iso_(Co6b).

The new ternary arsenide U_8_Co_42_As_25_ is isotypic with Y_8_Co_41_As_25_.^44^ The structure belongs to a large group of ternary compounds with a metal-to-metaloid ratio close to 2:1. Typically for this family, all atoms are located on two planes separated by half of the short translation period ∼4 Å. For better visualization, these structures are frequently described as an arrangement of columns, consisting of trigonal prisms formed by metal elements around metaloid atoms. Such a description might not reflect the real bonding situation, but is convenient for the visualization of the structures and their systematization,^48–50^ as well as for the prediction of the missing members of the respective series.^51^

In the U_8_Co_42_As_25_ structure, the trigonal prisms [U_2_Co_4_] around As atoms are condensed via common U atoms into a so-called “shamrock” fragment (Figure 3). Four individual “shamrock” segments are interconnected by sharing Co atoms into “propeller”-like agglomerates. The composition of the “propeller”-like agglomerates (one slab) can be written as U_4_Co_21_As_12_ (1 × UCo_3_Co_3/2_As_3_ + 3 × UCo_5_Co_1/2_As, where Co_3/2_ and Co_1/2_ are shared cobalt atoms of central and peripheral fragments, respectively). The neighboring agglomerates are shifted by *c*/2, relative to each other—see Figure 3 (Extended image version of the U_8_Co_42_As_25_ structure can be found in Figure S1). As a separate entity, the “shamrock” fragments can be distinguished in structures related to Zr_2_Fe_12_P_7_,^52^ as was recently discussed in the case of Th_2_Fe_12_As_7_.^45^

**Figure 3:**
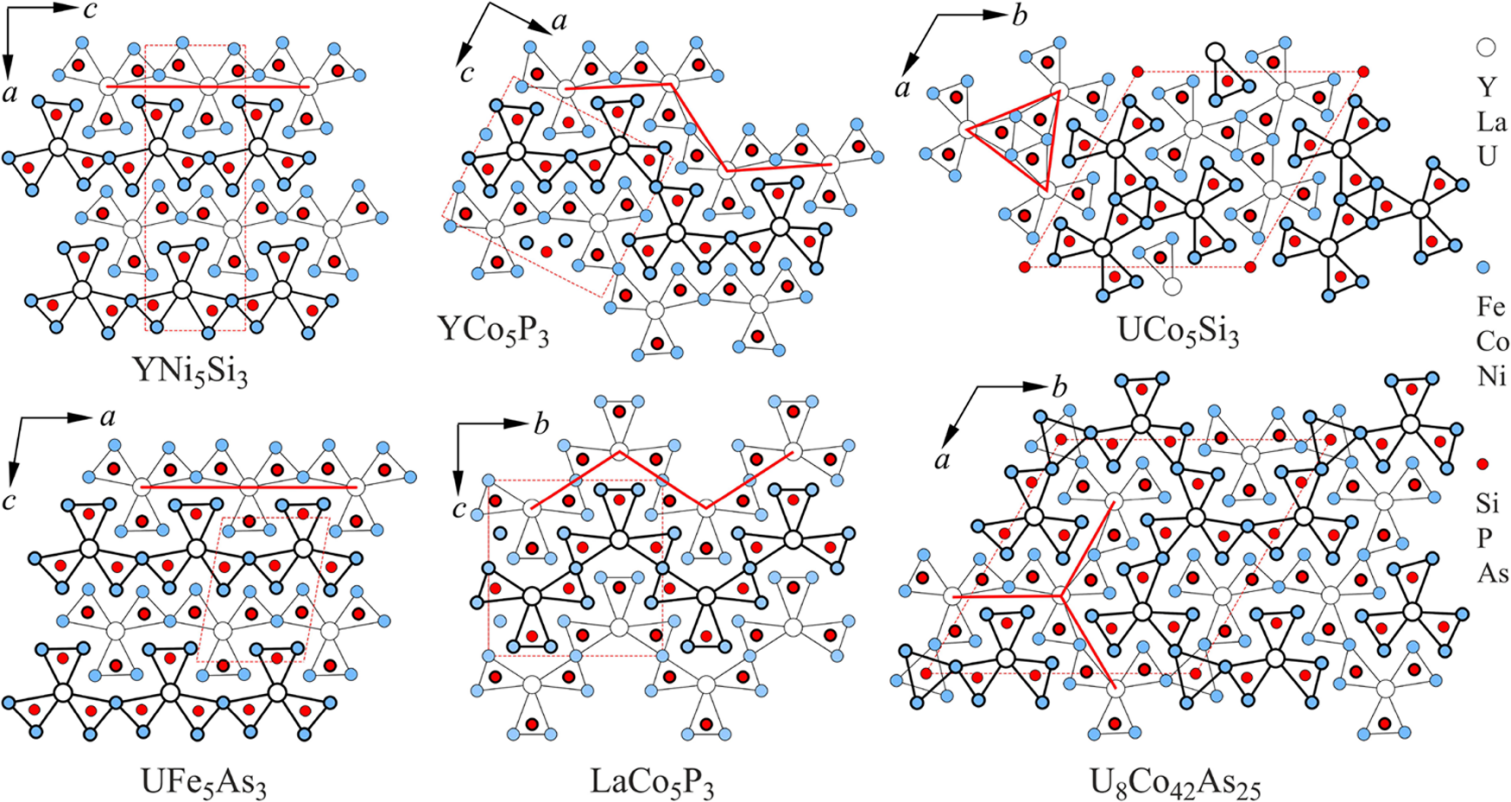
Crystal structures of 1:5:3 compounds that are described by connecting “shamrocks” in a straight line (YNi_5_Si_3_ and UFe_5_As_3_), a zigzag chain (YCo_5_P_3_ and LaCo_5_P_3_) or islands (UCo_5_Si_3_ and U_8_Co_42_As_25_). All atoms are located on two parallel planes, highlighted by thin and thick lines, respectively. Red dashed lines highlight unit cells for each of the structures. For better visualization of the relative arrangement of “shamrock” fragments, their centers are connected by solid red lines. For a complete structural representation of U_8_Co_42_As_25_, see Figure S1.

In addition to the reported U_8_Co_42_As_25_, a relatively large group of structures with a composition close to 1:5:3 can be also described with interconnected “shamrocks”: UFe_5_As_3_,^53^ LaCo_5_P_3_,^54^ YNi_5_Si_3_,^55^ YCo_5_P_3_,^56^ and UCo_5_Si_3_ ^57^—as shown in Figure 3. This kind of representation originates from Zr_2_Fe_12_P_7_.^52^ The latter are related to the Yb_6_Co_30_P_19_ structure^58^ and differ from it in that they lack atoms on the [001] axes. To better visualize the relative arrangement of the “shamrock” fragments, we connect their centers (uranium or rare earth atoms) to each other. Thus, three groups of structures can be distinguished, depending on the interconnection pattern of “shamrocks”: linear, zigzag or island-like. The crystal structures of the first group, YNi_5_Si_3_ and UFe_5_As_3_, differ in the relative location of every second linear block of condensed “shamrocks”. While the position of these blocks is identical in YNi_5_Si_3_, they are shifted along the [100] direction in the structure of UFe_5_As_3_, which requires monoclinic symmetry for structure description. For a better comparison of the second group, the structure of YCo_5_P_3_ is rotated so that the individual “shamrock” fragments have a comparable orientation with non-shared trigonal prisms. In the structure of LaCo_5_P_3_, each zigzag arrangement is formed by alternating inverted “shamrock” fragments. In the case of YCo_5_P_3_, all fragments within the same zigzag chain maintain their orientation. In contrast to the structures just discussed, the arrangement of the “shamrocks” in the third group is not infinite. They are grouped together in triangular blocks, formed by three (UCo_5_Si_3_) or four “shamrocks” (U_8_Co_42_As_25_). The structure of UCo_5_Si_3_ is the only one in the discussed 1:5:3 series in which such agglomerates are formed across two edges of “shamrocks”.

In principle, the relative arrangement of the “shamrock” fragments should be the origin of magnetic behavior of these materials. In previously reported UFe_5_As_3_ (*T*_*N*_ = 47 K, θ_*W*_ = −57 K^53^), the “shamrocks” form a line, resulting in a linear arrangement of the magnetic species. As a result, UFe_5_As_3_ does not show any signs of frustration. On the other hand, “shamrock” blocks form a 2D arrangement at *z* = 3/4 and island-like fragments at *z* = 1/4 due to the formation of the trigonal prism [As_5_Co_6_] in U_8_Co_42_As_25_ (*T*_*N*_ = 26 K, θ_*W*_ = −70 K), which could result in some magnetic frustration. An analysis of the “in-between” zigzag arrangement with compounds containing uranium would therefore be of interest.

The shortest uranium–uranium distance in the U_8_Co_42_As_25_ structure is *d*_U–U_ = 3.8120(1) Å (see Table S3). It is rather large, in comparison with those found in elemental uranium (*d*_U–U_ = 2.75–3.43 Å).^59^ According to the Hill limit,^60^ such a large separation is likely to yield a magnetic ground state, which is indeed seen in U_8_Co_42_As_25_. The shortest U–Co and U–As contacts are 2.9836(7) Å and 2.9287(6) Å, respectively. This is significantly larger, compared to the sum of covalent radii *r*_U_ + *r*_Co_ = 2.58 Å and *r*_U_ + *r*_As_ = 2.63 Å. The Co–As contacts between 2.273(4) Å and 2.445(2) Å are close to the sum of the covalent radii of these elements (*r*_Co_ + *r*_As_ = 2.37 Å). Thus, the crystal structure of U_8_Co_42_As_25_ can be described alternatively as a three-dimensional polyanionic Co–As-framework with embedded U atoms, similar to the Th_2_Fe_12_As_7_ compound.^45^

The crystal structure of UCo_3_As_2_ adopts the HoCo_3_P_2_ type (space group *Pmmn*).^61^ As is typical for members of the family with a metal–metalloid ratio of 2:1, most atoms in the structure (with the exception of the Co6b position) are located on two mirror planes at *x* = 1/4 and 3/4. The structure can be described as an arrangement of trigonal blocks, whereby a central trigonal prism of U atoms around the Co position is extended by six additional prisms around As atoms: [U_4_Co_2_] (3×) and [U_2_Co_4_] (3×), which are attached to rectangular faces and lateral edges, respectively (see Figure 4).

**Figure 4:**
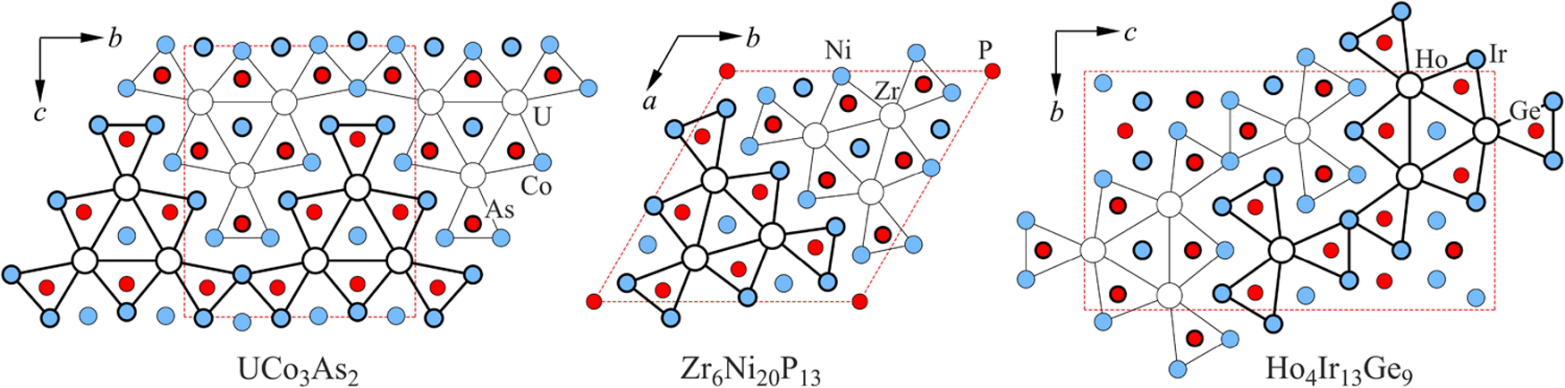
Crystal structures of UCo_3_As_2_, Zr_6_Ni_20_P_13_, and Ho_4_Ir_13_Ge_9_, represented as condensed trigonal prisms around nonmetal atoms.

The blocks—we will refer to these as “1 + 6” for the rest of the discussion—are connected to each other via shared edges to form infinite chains along the [010] direction. As a separate unit, such a block can be observed in the Zr_6_Ni_20_P_13_ structure,^62^ see Figure 4. These building units, in combination with other blocks, can also be distinguished in other structures of the discussed 2:1 series, e.g., Ce_9_Ni_26_P_12_.^63^ However, taking into account compounds of this manuscript, the Ho_4_Ir_13_Ge_9_ ^64^ and Zr_4_Co_13_Si_9_ ^65,66^ structures are worth mentioning, as they contain both structural fragments of U_8_Co_42_As_25_ and UCo_3_As_2_. In the structure of Zr_4_Co_13_Si_9_, the individual “shamrocks” are embedded into a matrix of interconnected “1 + 6” blocks. In contrast, for Ho_4_Ir_13_Ge_9_, both units are connected only by a shared edge, as can be seen from the Figure 4 (right panel).

Analysis of the interatomic distances in the UCo_3_As_2_ structure shows a similar trend to U_8_Co_42_As_25_:

(i) absence of short As–As contacts;
(ii) long U–U distances – 3.8611(2) Å along [100] and 4.271(2) Å within the “1 + 6” block;
(iii) significantly larger U–Co (2.963(3) Å) and U–As (2.9232(6) Å) distances, compared to the sum of respective single bond radii (see Figure 2b);
(iv) short Co–As contacts of 2.280(3)–2.407(8) Å.

The only exception is the U–Co6b contacts, which are located outside the mirror plane – see Figure 2b. The shortest distances U1–Co6b and U2–Co6b of 2.784(6) Å and 2.747(7) Å, respectively, can be explained by different oxidation states of Co6b and Co6a species. The alternative occupation of Co6b position by As atoms cannot be ruled out, but is difficult to estimate based on the X-ray data.

Temperature-dependent magnetic susceptibilities for U_8_Co_42_As_25_ and UCo_3_As_2_ are shown in Figure 5. Both compounds exhibit antiferromagnetic transitions with *T*_*N*_ = 26 K (U_8_Co_42_As_25_) and *T*_*N*_ = 67 K (UCo_3_As_2_). For UCo_3_As_2_, the difference between the magnetic susceptibility for μ_0_ *H* = 3.5 T and μ_0_ *H* = 7 T can possibly be attributed to the presence of a small (ppm) amount of ferromagnetic impurities, which may originate from the starting elements. The ferromagnetic impurity contribution was subtracted by using the Honda-Owen method.^67,68^ The inverse susceptibility was plotted vs. temperature and fit with the Curie–Weiss law for temperatures above 100 K, yielding μ_eff_ = 20.4 μ_B_ per formula unit (F.U.) and Θ_W_ = −70 K for U_8_Co_42_As_25_. For UCo_3_As_2_, the values of μ_eff_ = 2.01 μ_B_ per F.U. and Θ_W_ = 1 K were obtained. While the sharp transition observed in U_8_Co_42_As_25_ likely reflects the similar coordination environment of U1 and U2 atoms, meanwhile the broad transition in UCo_3_As_2_ may be understood as caused by large differences in the coordination of U2 atoms, caused by the observed disorder. For both compounds, the exact magnetic configuration remains to be determined and will be topic of a future study.

**Figure 5:**
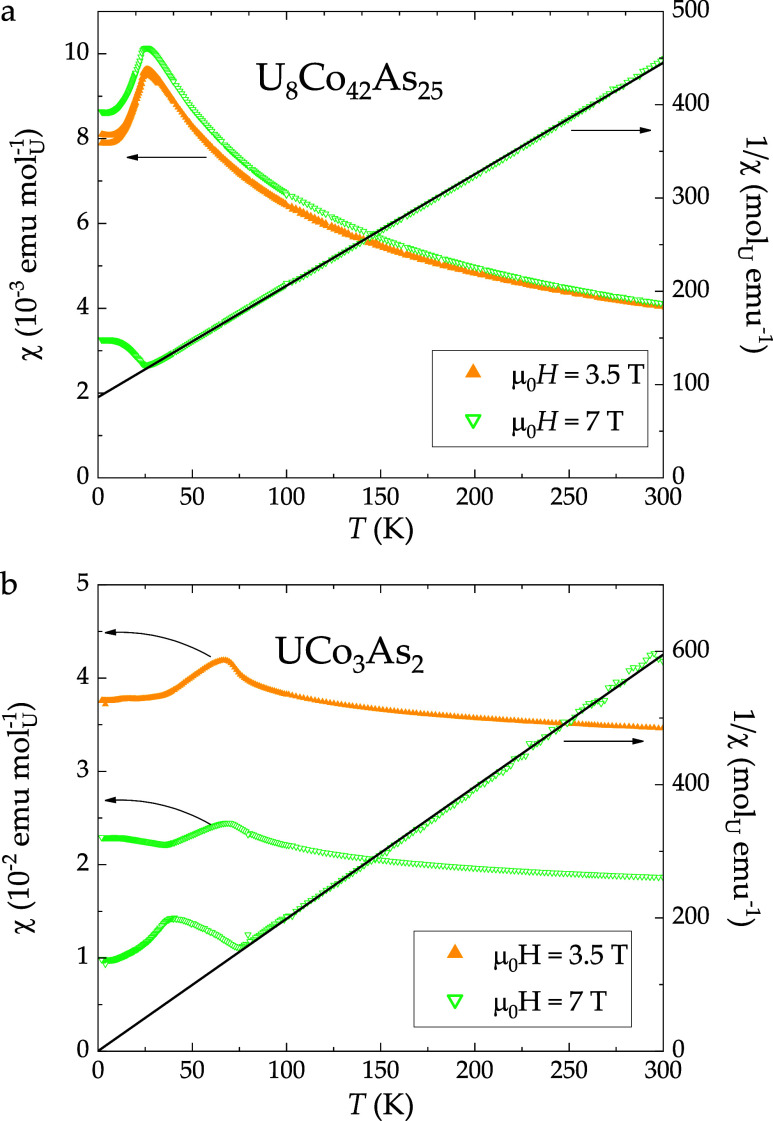
Magnetic susceptibilities of (a) U_8_Co_42_As_25_ and (b) UCo_3_As_2_ shows features characteristic of an antiferromagnetic ordering below *T*_*N*_ = 26 K and *T*_*N*_ = 67 K, respectively.

Consistent with magnetization data, the temperature dependence of specific heat, presented in Figure 6, confirms bulk magnetic transitions at *T*_*N*_ = 26 K (U_8_Co_42_As_25_) and *T*_*N*_ = 67 K (UCo_3_As_2_). From the *C*_*p*_/*T* vs *T* ^2^ data at the lowest temperature the value of the Sommerfeld coefficient γ_0_ can be estimated. Since both compounds have fairly high ordering temperatures, the Sommerfeld coefficient values of 155 mJ mol_U_^–1^ K^–1^ (U_8_Co_42_As_25_) and 95 mJ mol_U_^–1^ K^–1^ (UCo_3_As_2_) can be an overestimate (the fit are shown in the insets of Figure 6). While these compounds fulfill some of the empirical factors previously suggested to lead to heavy-Fermion behavior in uranium-based systems,^8^ further studies on U_8_Co_42_As_25_ and UCo_3_As_2_ are needed. An enhanced value of γ_0_ is, in principle, consistent with the Kondo-like shape of the electrical resistivity, shown in Figure 6. Due to the relatively small size of the single crystals for both compounds, microscale devices were fabricated using FIB in order to examine their electrical resistivity. Examples of the microscaled devices are shown in Figure 1, where the electric current was applied along the [001] (U_8_Co_42_As_25_) or [100] (UCo_3_As_2_) axis. The magnetic transitions at *T*_*N*_ = 26 K (U_8_Co_42_As_25_) and *T*_*N*_ = 67 K (UCo_3_As_2_) are marked by corresponding features in electrical resistivity, with both materials having relatively low residual resistivity ratios RRR = 5.0 (U_8_Co_42_As_25_) and RRR = 1.6 (UCo_3_As_2_), consistent with structural disorder described above. The value of the Kadowaki-Woods ratio^69,70^ determined as *A*/γ^2^ (where *A* is the quadratic coefficient of the low-temperature electrical resistivity—see the insets of Figure 6) amounts to 2 μΩ cm mol_U_^2^K^2^J^–2^(U_8_Co_42_As_25_) and 8 μΩ cm mol_U_^2^K^2^J^–2^ (UCo_3_As_2_). Both values are significantly different from the Kadowaki-Woods ratios of the exceptionally heavy uranium-based heavy-Fermion compounds such as UBe_13_,^71^ UCd_11_,^72,73^ U_23_Hg_88_ ^3^ and U_2_Zn_17_.^74^

**Figure 6:**
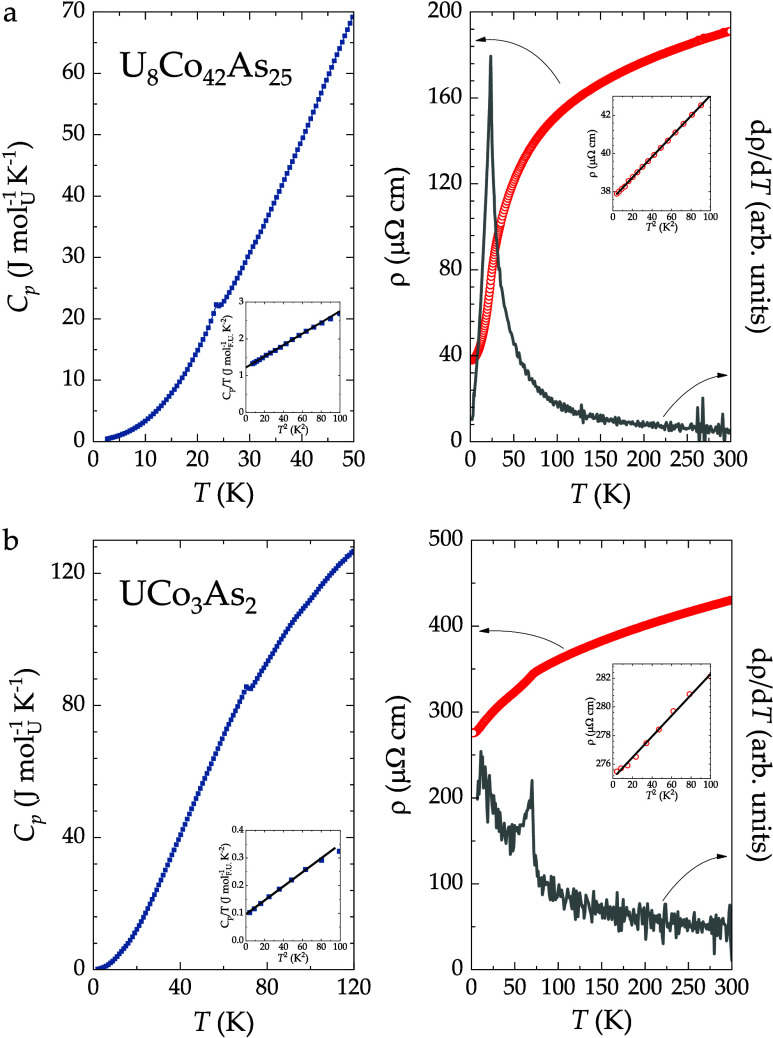
Specific heat and electrical resistivity of (a) U_8_Co_42_As_25_ and (b) UCo_3_As_2_. Insets show the *C*_*p*_/*T* (or ρ) vs *T* ^2^ data from which γ (or *A*) coefficient is extracted. The derivative of the resistivity data is used to pinpoint the magnetic order. Magnetic transitions, observed at *T*_*N*_ = 26 K (U_8_Co_42_As_25_) and *T*_*N*_ = 67 K (UCo_3_As_2_) are confirmed by both specific heat and transport data.

## 4 Conclusions

In this work, we successfully revisit the Co-rich part of the uranium-cobalt-arsenic ternary system, where only two compounds U_2_Co_12_As_7_ and UCoAs_2_ were known prior to our investigation. Applying the bismuth flux technique, we were able to synthesize two new magnetic systems—U_8_Co_42_As_25_ and UCo_3_As_2_. Both structures belong to the large group of intermetallic compounds with a metal-to-metalloid ratio 2:1 and trigonal prismatic coordination of the main group element. A transition into the antiferromagnetic state is observed at *T*_*N*_ = 26 K for U_8_Co_42_As_25_ and *T*_*N*_ = 67 K for UCo_3_As_2_. A more precise analysis of the exact magnetic configuration as well as oxidation states of both U and Co should be possible by an XMCD/XANES analysis, which will be a topic of a future study.

## Supplementary Material



## Notes

Further structural representation of the title compounds, together with additional crystallographic data (PDF)
