# Supplementary material for: Antiferromagnetic Arsenides U8Co42As25 and UCo3As2
Source: Inorg Chem. 2026 Jul 11;65(29):16774–82. doi: 10.1021/acs.inorgchem.6c01592 (PMC13418166; doi:10.1021/acs.inorgchem.6c01592)
Supplement: Supplementary file 1 [file ic6c01592_si_001.pdf]

Supporting Information

Antiferromagnetic arsenides $U_8Co_{42}As_{25}$ and UCo_3As_2

Nazar Zaremba,[1] Mitja Krnel,[1] Yurii Prots,[1] Orest Pavlosiuk,[2] Lev Akselrud,[3] Andreas Leithe-Jasper,[1] Markus König,[1] Yuri Grin,[1] and Eteri Svanidze,[1,*]

[1]*Max Planck Institute for Chemical Physics of Solids, 01187 Dresden, Germany*

[2]*Institute of Low Temperature and Structure Research, Polish Academy of Sciences, 50-422 Wrocław, Poland*

[3]*Department of Inorganic Chemistry, Ivan Franko National University of Lviv, 79000 Lviv, Ukraine*

[*]*Eteri.Svanidze@cpfs.mpg.de*

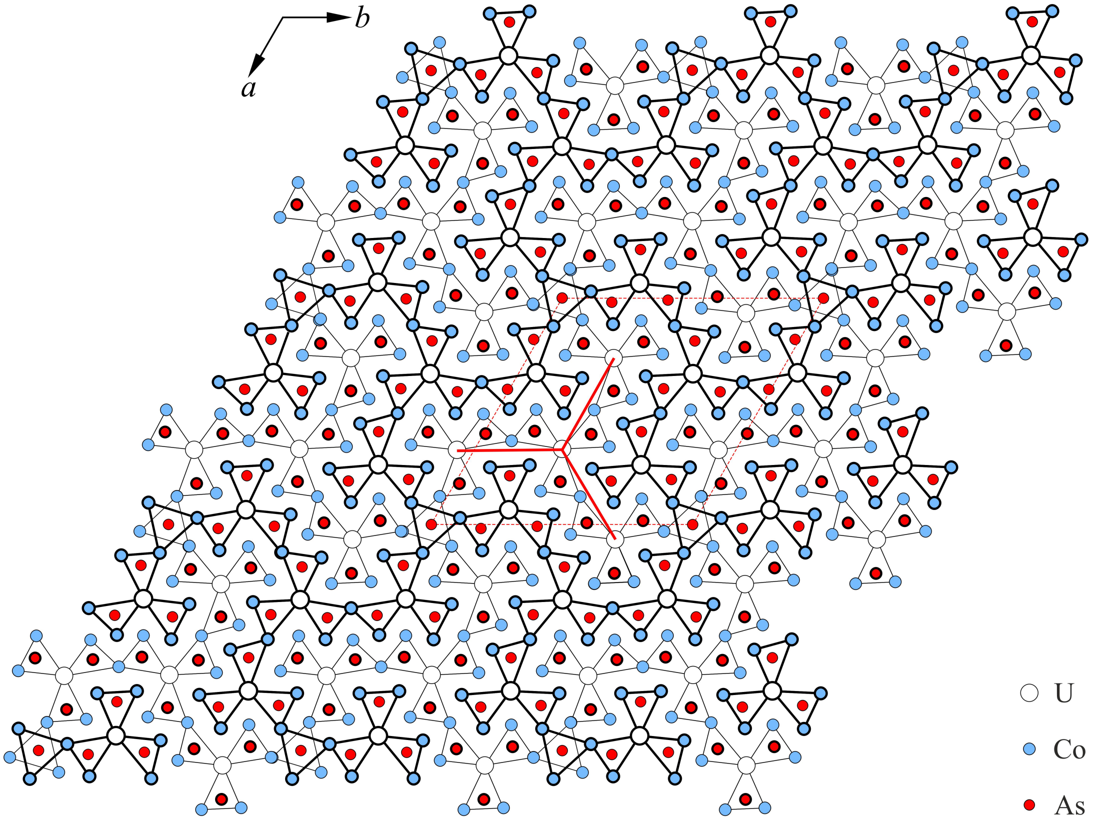

$U_8Co_{42}As_{25}$

Figure S1. Extended version of the $U_8Co_{42}As_{25}$ structure.

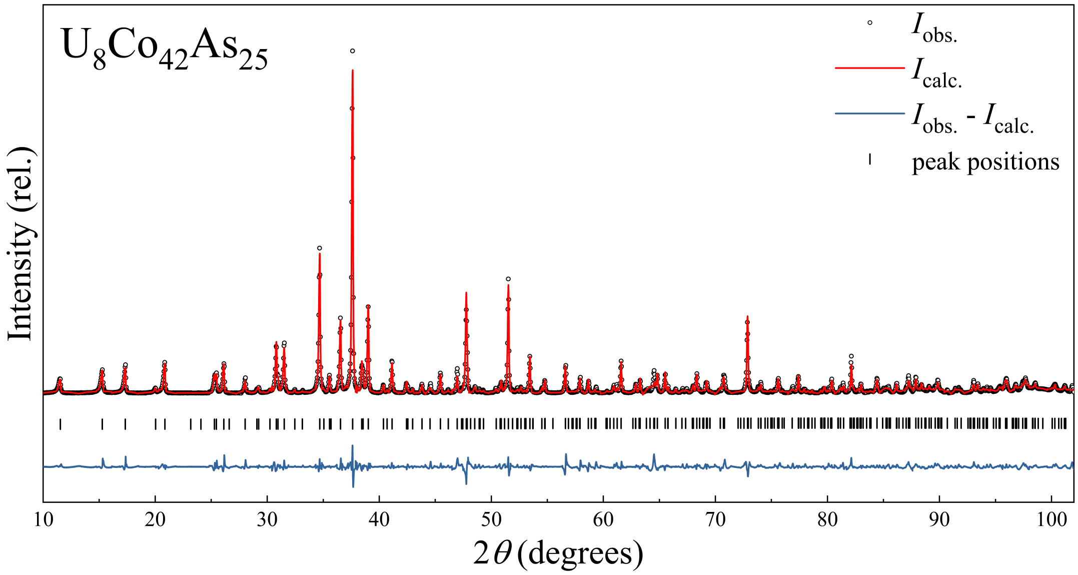

Figure S2. Powder XRD pattern (CuKα_1 radiation, λ = 1.54056 Å) for $U_8Co_{42}As_{25}$ ($I_{obs.}$, symbols), together with the calculated profile ($I_{calc.}$, red line), difference between them ($I_{obs.}$ - $I_{calc.}$, blue line), and calculated positions of the Bragg reflections (vertical ticks).

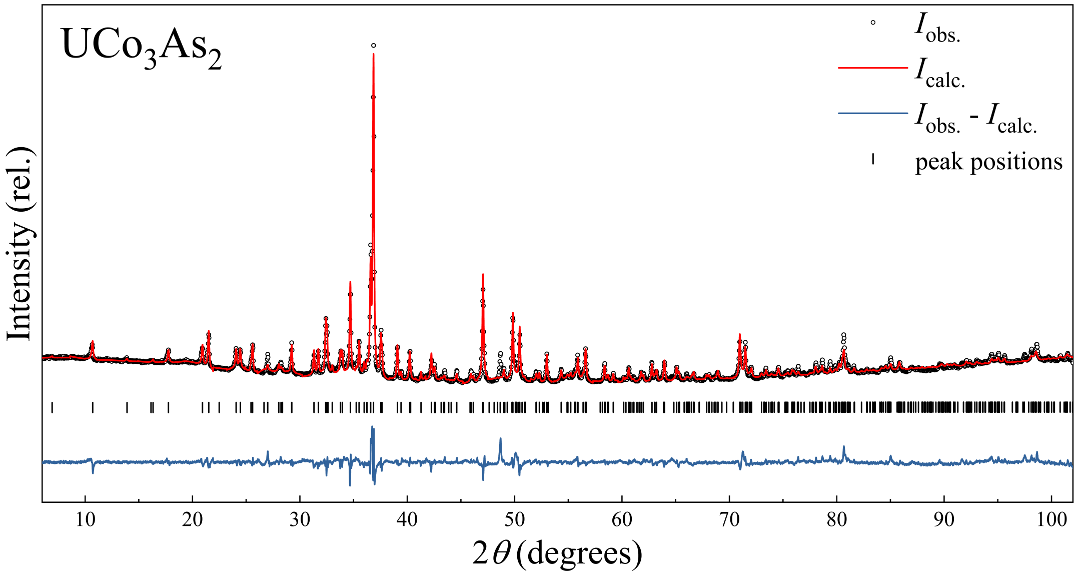

Figure S3. Powder XRD pattern (CuKα_1 radiation, λ = 1.54056 Å) of UCo_3As_2 ($I_{obs.}$, symbols), together with the calculated profile ($I_{calc.}$, red line), difference between them ($I_{obs.}$ - $I_{calc.}$, blue line), and calculated positions of the Bragg reflections (vertical ticks).

Table S1. Anisotropic displacement parameters (Å^2) for $U_8Co_{42}As_{25}$ structure; U_{23} = U_{13} = 0.

Atom	U_{11}	U_{22}	U_{33}	U_{12}
U1	0.00922(11)	0.00818(11)	0.00661(9)	0.00426(9)
U2	0.00870(12)	0.00870(12)	0.00651(18)	0.00435(6)
Co1	0.0076(4)	0.0080(4)	0.0072(4)	0.0035(3)
Co3	0.0110(4)	0.0098(4)	0.0081(4)	0.0063(4)
Co4	0.0091(4)	0.0099(4)	0.0072(4)	0.0057(4)
Co5	0.0091(4)	0.0078(4)	0.0089(4)	0.0032(3)
Co6	0.0083(4)	0.0076(4)	0.0070(4)	0.0044(3)
Co7	0.0134(5)	0.0090(4)	0.0117(4)	0.0057(4)
As1	0.0079(3)	0.0077(3)	0.0070(3)	0.0044(2)
As2	0.0082(3)	0.0088(3)	0.0070(3)	0.0042(2)
As3	0.0066(3)	0.0085(3)	0.0072(3)	0.0039(3)
As4	0.0090(3)	0.0078(3)	0.0068(3)	0.0047(3)
As5	0.0069(6)	0.0069(6)	0.0126(12)	0.0034(3)

Table S2. Anisotropic displacement parameters (Å^2) for UCo_3As_2 structure; U_{13} = U_{12} = 0.

Atom	U_{11}	U_{22}	U_{33}	U_{23}
U1	0.00526(14)	0.00842(14)	0.00751(15)	0
U2	0.00498(11)	0.00888(11)	0.00637(11)	-0.00002(7)
As1	0.0055(3)	0.0071(3)	0.0052(3)	-0.0002(2)
As2	0.0056(3)	0.0080(3)	0.0055(3)	0.0006(2)
As3	0.0054(4)	0.0068(4)	0.0053(4)	0
As4	0.0054(4)	0.0082(4)	0.0066(4)	0
Co1	0.0126(6)	0.0122(6)	0.0095(6)	0
Co2	0.0057(5)	0.0078(5)	0.0071(5)	0
Co3	0.0061(4)	0.0095(4)	0.0063(4)	0.0003(3)
Co4	0.0061(4)	0.0065(4)	0.0082(4)	-0.0010(3)
Co5	0.0080(4)	0.0077(4)	0.0073(4)	0.0005(3)

Table S3. Interatomic distances in $U_8Co_{42}As_{25}$.[a]

Atoms		δ, Å	Atoms		δ, Å	Atoms		δ, Å	Atoms		δ, Å
U1–	2As1	2.943(1)	Co1–	1As3	2.274(1)	Co4–	1As3	2.344(2)	As1–	1Co3	2.297(2)
	2As2	2.972(1)		1As1	2.336(2)		1As3	2.346(1)		1Co1	2.336(2)
	2Co5	2.984(1)		2As1	2.337(1)		2As4	2.445(1)		2Co1	2.337(1)
	2As4	2.992(1)		2Co1	2.617(2)		2Co6	2.691(2)		1Co6	2.399(1)
	1Co1	3.046(1)		2Co6	2.631(1)		2Co7	2.809(2)		2Co7	2.638(1)
	1Co2b	3.093(3)		1Co7	2.641(2)		2U2	3.104(1)		2U1	2.943(1)
	2Co6	3.142(1)		2Co7	2.897(1)		1U1	2.330(1)	As2–	1Co2b	2.284(4)
	2Co3	3.169(1)		1U1	3.046(1)	Co5–	1As2	2.396(2)		1Co3	2.351(1)
	1Co5	3.297(2)	Co2a[a]–	1Co2b	0.512(3)		1As4	2.397(1)		2Co2b	2.371(2)
	1Co4	3.330(1)		1As5	1.752(3)		2As2	2.414(1)		1Co5	2.396(2)
	1Co2a	3.603(3)		1As2	2.415(3)		1Co2b	2.593(3)		2Co5	2.414(1)
	1Co7	3.756(2)		2As5	2.598(2)		2Co2b	2.700(2)		2Co2a	2.612(2)
	1As2	3.776(1)		4Co2a	2.598(2)		2Co3	2.707(1)		2U1	2.972(1)
	2U1	3.8120(1)		2As2	2.612(2)		1Co2a	2.758(3)	As3–	1Co1	2.274(1)
	1As3	3.970(1)		1Co5	2.758(3)		2U1	2.984(1)		1Co4	2.344(2)
	1As4	3.975(1)		2Co2b	2.788(4)		2Co2a	3.005(3)		1Co4	2.346(1)
U2–	4As3	2.929(1)		2Co2b	2.819(4)		1U1	3.297(2)		2Co6	2.410(8)
	1Co4	3.104(1)		2Co5	3.005(3)		2Co3	2.707(1)		2Co7	2.549(1)
	5Co4	3.105(1)		2Co2a	3.034(4)		1Co2a	2.758(3)		2U2	2.929(1)
	3Co6	3.205(1)	Co2b[a]–	1Co2a	0.512(3)		2U1	2.984(1)	As4–	1Co6	2.340(1)
	3Co7	3.462(1)		1As5	2.263(3)		2Co2a	3.005(3)		1Co7	2.364(1)
	1U2	3.8120(1)		1As2	2.284(4)		1U1	3.297(2)		2Co3	2.393(1)
	1As4	3.981(1)		2As2	2.371(2)	Co6–	1As4	2.340(1)		1Co5	2.397(1)
	1As4	3.982(1)		1Co5	2.593(3)		1As1	2.399(1)		2Co4	2.445(1)
				2Co5	2.700(2)		2As3	2.410(8)		2U1	2.992(1)
				2Co2a	2.788(4)		2Co1	2.631(1)	As5[a]–	2Co2a	1.752(3)
				2Co2a	2.819(4)		2Co4	2.691(2)		2As5	1.906(1)
				2Co2b	2.959(4)		1Co7	2.712(2)		1Co2b	2.263(3)
				2As5	2.959(2)		2U1	3.142(1)		2Co2a	2.598(2)
				1U1	3.093(3)		1U2	3.205(1)		2Co2b	2.959(2)
			Co3–	1As1	2.297(2)	Co7–	1As4	2.364(1)			
				1As2	2.351(1)		2As3	2.549(1)			
				2As4	2.393(1)		2As1	2.638(1)			
				1Co4	2.691(2)		1Co1	2.641(2)			
				2Co5	2.707(1)		1Co6	2.712(2)			
				2Co7	2.993(1)		2Co4	2.809(2)			
				1U1	3.141(1)		2Co1	2.897(1)			
				2U1	3.169(1)		2Co3	2.993(1)			
				1Co4	2.691(2)						
				2Co5	2.707(1)						
				2Co7	2.993(1)						
				1U1	3.143(2)						
				2U1	3.169(1)						

[a] Atoms Co2a, Co2b, and As5 are partially occupied. Some of the distances do not reflect real contact, but distances between calculated positions.

Table S4. Interatomic distances in UCo_3As_2. [a]

Atoms		δ, Å	Atoms		δ, Å	Atoms		δ, Å	Atoms		δ, Å
U1–	2Co6b	2.747(7)	Co1–	1As4	2.396(2)	Co4–	1As3	2.349(1)	As1–	1Co6a	2.305(2)
	2Co6a	2.963(2)		2As2	2.610(1)		1As2	2.364(1)		2Co6b	2.336(3)
	2As4	2.989(1)		2As2	2.611(1)		2As2	2.407(1)		1Co3	2.352(1)
	4As1	3.028(1)		2Co4	2.727(2)		2Co4	2.634(1)		2Co3	2.365(1)
	4Co3	3.122(1)		4Co5	2.910(2)		2Co2	2.657(1)		1Co5	2.395(1)
	2Co6b	3.213(7)		2Co2	2.943(2)		1Co1	2.727(2)		2U2	2.982(1)
	2Co5	3.227(1)	Co2–	2As2	2.309(1)		1U2	3.146(1)		2U1	3.028(1)
	2Co3	3.334(1)		2As3	2.329(1)		2U2	3.238(1)	As2–	1Co2	2.309(1)
	2Co5	3.227(1)		4Co4	2.657(1)	Co5–	1As2	2.361(1)		1Co5	2.361(1)
	2Co3	3.334(1)		2Co1	2.943(2)		1As1	2.395(1)		1Co4	2.364(1)
U2–	2Co6b	2.784(6)		2U1	3.115(1)		2As4	2.430(2)		2Co4	2.407(1)
	2As2	2.923(1)	Co3–	1As1	2.352(1)		1Co5	2.637(4)		2Co1	2.611(1)
	2As3	2.976(1)		2As1	2.365(1)		2Co3	2.731(1)		2U2	2.923(1)
	2As1	2.982(1)		1As4	2.400(1)		2Co1	2.910(2)	As3–	1Co6a	2.280(3)
	2Co6a	3.004(1)		2Co5	2.731(1)		2U2	3.065(1)		2Co6b	2.302(6)
	2Co5	3.065(1)		2Co3	2.874(1)		1U1	3.227(1)		2Co2	2.329(1)
	1Co3	3.108(1)		1U2	3.108(1)	Co6a[a]–	2Co6b	0.359(8)		2Co4	2.349(1)
	1Co2	3.115(1)		2U1	3.122(1)		1As3	2.280(3)		4U2	2.976(1)
	1Co4	3.146(1)		1U1	3.334(1)		1As1	2.305(2)	As4–	1Co1	2.396(2)
	2Co4	3.238(1)					1As1	2.306(1)		2Co3	2.400(1)
	2Co6b	3.244(6)					2U1	2.963(2)		4Co5	2.430(2)
	1Co1	3.506(1)					4U2	3.004(1)		2U1	2.989(1)
						Co6b[a]–	1Co6a	0.359(8)			
							1Co6b	0.718(1)			
							1As3	2.302(6)			
							2As1	2.336(3)			
							1U1	2.747(7)			
							2U2	2.784(6)			
							1Co6b	3.143(1)			
							1U1	3.213(7)			
							2U2	3.244(6)			

[a] Atoms Co6a and Co6b are partially occupied. Some of the distances do not reflect real contact, but distances between calculated positions.
